# Complete chloroplast of *Micractinium pusillum* CCAP 231/1 (chlorellaceae, trebouxiophyceae)

**DOI:** 10.1080/23802359.2019.1698342

**Published:** 2019-12-13

**Authors:** Kyeong Mi Kim, Seung-Woo Jo, Nam Seon Kang, Jung A. Lee, Eun Song Kim, Moongeun Yoon, Hyeong Seok Jang, Ji Won Hong, Ho-Sung Yoon

**Affiliations:** a Department of Taxonomy and Systematics, National Marine Biodiversity Institute of Korea, Seocheon, Republic of Korea;; b Department of Energy Science, Kyungpook National University, Daegu, Republic of Korea;; c School of Life Sciences, BK21 Plus KNU Creative BioResearch Group, Kyungpook National University, Daegu, Republic of Korea

**Keywords:** Complete chloroplast genome, *Micractinium pusillum*, Chlorellaceae, Trebouxiophyceae

## Abstract

The chloroplast genome of *Micractinium pusillum* CCAP 231/1 was completely sequenced. This chloroplast genome has 115,638 bp in length and consists of 111 genes including 81 protein-coding, 4 rRNA, and 26 tRNA genes. The overall GC content of the genome is 35.3%.

*Micractinium* Fresenius is a cosmopolitan genus commonly found in freshwater environments. Currently, there are 16 species that have been taxonomically accepted belonging the genus *Micractinium* (Guiry and Guiry 2019). *Micractinium pusillum* Fresenius (Chlorellaceae, Trebouxiophyceae) is a holotype species of the genus. This species is known to be distributed in various freshwater habitats (John et al. 2002). In this study, the complete chloroplast of *M*. *pusillum* CCAP 231/1 was determined for the first time.

*M*. *pusillum* CCAP 231/1 was obtained from the Culture Collection of Algae and Protozoa (CCAP) and this strain has a British origin (Wicken Lode, Cambridgeshire, England, 52°18′ 24.29″N 0°16′32.86″E). The culture was grown in BG-11 medium (UTEX, USA) containing imipenem (Sigma-Aldrich, USA) at a concentration of 100 µg mL^−1^ to eliminate bacterial growth (Hong et al. 2015) and grown at 18 °C under cool fluorescent light (approximately 40 µmole m^−2^ s^−1^) in a light:dark cycle (14:10 h) for 4 weeks. Microalgal biomass was collected by centrifugation at centrifugation at 2063 ×*g* (1580 R; Labogene, Korea). Whole genomic DNA was extracted from the sample using a DNeasy Plant Mini Kit (Qiagen, Germany) followed by preparation of a library using an MGIEasy DNA Library Prep Kit V1 (BGI, China) according to the manufacturer’s instruction. Whole genome sequencing was performed using BGISEQ-500 (BGI, China) sequencer and raw data was filtered to obtain >10 Gb clean data per each sample. *De novo* plastome assembly was carried out using NOVOPlasty v3.6 software (Dierckxsens et al. 2017). The size of the circular plastome produced is 115,638 bp (GenBank accession number MN649872) which is smaller than that of the previously reported *M*. *conductrix* (149,364 bp, GenBank accession number KY629619). The nucleotide composition is 32.6% A, 32.2% T, 17.2% G, and 18.1% C. The overall GC content is 35.3%. The *M*. *pusillum* chloroplast genome contains 111 genes, including 81 predicted protein-coding, 4 rRNA, and 26 tRNA genes. Among 81 genes, 61 genes were revealed as complete protein-coding genes, which 60 genes were started with ATG and 1 gene was with GTG as a start codon and all of these 61 genes were ended with TAA as a stop codon. It was found that there were 5 cases of gene-overlapping ranging from 44 to 9424 bp in size. All 26 tRNA genes ranged from 48 to 87 bp in length.

Phylogenetic analysis was carried out by PhyML 3.0 with 13 reported plastome sequences (Fan et al. 2017) belonging to the Trebouxiophyceae family and the result was visualized by FigTree v1.4.4. (Figure 1). It showed the phylogenetic position of *M*. *pusillum* CCAP 231/1 (Figure 1) within the family. This new information would contribute to the better understanding of the phylogenetic relationships of the *Micractinium* species and chloroplast genome diversity and evolution in the Trebouxiophyceae.

**Figure 1.**
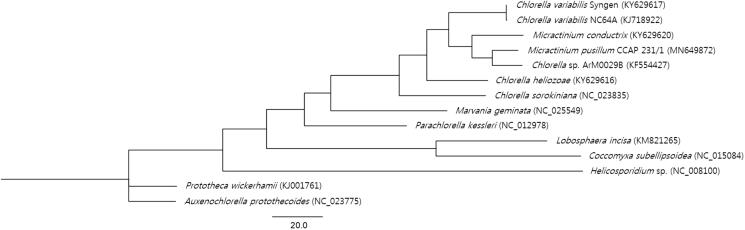
Maximum-likelihood phylogenetic tree of M. pusillum CCAP 231/1 and 13 other species. GenBank accession numbers were indicated in the parentheses.
